# 1681. Persistent Increased Invasive Pediatric *Streptococcus pyogenes* (Group A *Streptococcus*) Infections with Correlated Increase in Superficial Infection and Pharyngitis

**DOI:** 10.1093/ofid/ofad500.1514

**Published:** 2023-11-27

**Authors:** Jameson Dowling, Daphne Werz, Kenneth Smith, Rebecca Harris, Sarah Smathers, Cecelia Harrison-Long, Julia Sammons, Ericka Hayes

**Affiliations:** Children's Hospital of Philadelphia/Department of Infection Prevention & Control, Philadelphia, Pennsylvania; Children's Hospital of Philadelphia, Department of Infection Prevention & Control, Downingtown, Pennsylvania; Children's Hospital of Philadelphia, Philadelphia, Pennsylvania; Children's Hospital of Philadelphia, Philadelphia, Pennsylvania; Children's Hospital of Philadelphia, Philadelphia, Pennsylvania; Children's Hospital of Philadelphia, Philadelphia, Pennsylvania; Children's Hospital of Philadelphia, Philadelphia, Pennsylvania; Children's Hospital of Philadelphia, Philadelphia, Pennsylvania

## Abstract

**Background:**

Children's Hospital of Philadelphia (CHOP) clinicians observed an increase in invasive group A *Streptococcus* infections (iGAS) in November 2022. In December 2022, the Centers for Disease Control and Prevention published an investigation of increased iGAS. At CHOP iGAS remain increased, even after peak respiratory viral season. Our objective was to compare pre-pandemic and current case counts and to investigate if the increase in iGAS correlates with an increase in non-invasive GAS infections (nGAS).

**Methods:**

Data for positive *Streptococcus pyogenes* results at CHOP from January 2018 to April 2023 was collected from the electronic medical record (n=466). Results were classified by source and specimen type as pharyngitis, superficial, and invasive, then further classified as non-invasive (pharyngitis and superficial) or invasive. For infection events with multiple results only one result was included, giving preference to the most aggressive classification (invasive >pharyngitis >superficial). Fisher's exact test was used to assess association between the increase in nGAS and iGAS prior to and after the start of the COVID-19 pandemic (2018-2019 vs. 2021-2023). Fisher's exact test, with Monte Carlo simulation, was used to assess association between the increase in nGAS and iGAS infections by year.

**Results:**

There was a statistically significant association in the increase in pharyngitis, superficial, and invasive group A *Streptococcus* infections (GAS) (p-value < 0.0001) and in nGAS and iGAS (p-value = 0.0001), when comparing pre- and post-2020. We found statistically significant associations in the increase of pharyngitis, superficial, and invasive GAS cases (p-value = 0.0005) and of nGAS and iGAS cases (p-value = 0.0005).

**Figure:**
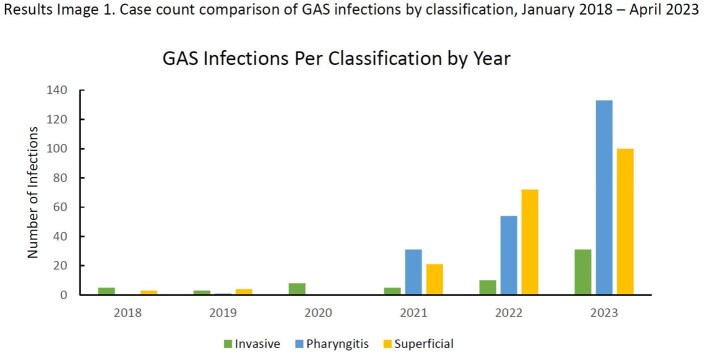

**Figure:**
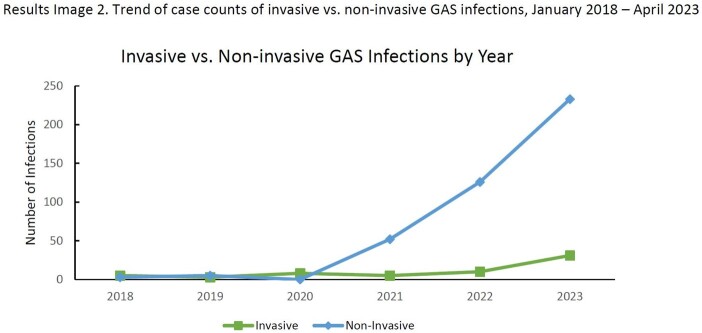

**Conclusion:**

Our data shows a significant increase in cases from pre-pandemic years for nGAS and iGAS infections. iGAS is correlated with nGAS, even with further stratification of nGAS into pharyngitis and superficial. Notably, increases in nGAS were significantly more than the increase in iGAS, which has unexpectedly persisted beyond the typical winter viral infection peak. Continued monitoring or GAS, particularly iGAS, is justified.

**Disclosures:**

**All Authors**: No reported disclosures

